# The paralog-to-contig assignment problem: high quality gene models from fragmented assemblies

**DOI:** 10.1186/s13015-016-0063-y

**Published:** 2016-02-24

**Authors:** Henrike Indrischek, Nicolas Wieseke, Peter F. Stadler, Sonja J. Prohaska

**Affiliations:** Computational EvoDevo Group, Department of Computer Science, Universität Leipzig, Härtelstraße 16–18, 04107 Leipzig, Germany; Bioinformatics Group, Department of Computer Science, Universität Leipzig, Härtelstraße 16–18, 04107 Leipzig, Germany; Interdisciplinary Center for Bioinformatics, Universität Leipzig, Härtelstraße 16–18, 04107 Leipzig, Germany; Parallel Computing and Complex Systems Group, Department of Computer Science, Universität Leipzig, Augustusplatz 10, 04109 Leipzig, Germany; Max Planck Institute for Mathematics in the Sciences, Inselstraße 22, 04103 Leipzig, Germany; Fraunhofer Institute for Cell Therapy and Immunology, Perlickstraße 1, 04103 Leipzig, Germany; Department of Theoretical Chemistry, University of Vienna, Währinger Straße 17, 1090 Vienna, Austria; Center for non-coding RNA in Technology and Health, Grønegårdsvej 3, 1870 Frederiksberg C, Denmark; Santa Fe Institute, 1399 Hyde Park Rd., Santa Fe, NM87501 USA

**Keywords:** Genome annotation, Gene models, Genome assembly, Assignment problems, Integer linear programming

## Abstract

**Background:**

The accurate annotation of genes in newly sequenced genomes remains a challenge. Although sophisticated comparative pipelines are available, computationally derived gene models are often less than perfect. This is particularly true when multiple similar paralogs are present. The issue is aggravated further when genomes are assembled only at a preliminary draft level to contigs or short scaffolds. However, these genomes deliver valuable information for studying gene families. High accuracy models of protein coding genes are needed in particular for phylogenetics and for the analysis of gene family histories.

**Results:**

We present a pipeline, ExonMatchSolver, that is designed to help the user to produce and curate high quality models of the protein-coding part of genes. The tool in particular tackles the problem of identifying those coding exon groups that belong to the same paralogous genes in a fragmented genome assembly. This paralog-to-contig assignment problem is shown to be NP-complete. It is phrased and solved as an Integer Linear Programming problem.

**Conclusions:**

The ExonMatchSolver-pipeline can be employed to build highly accurate models of protein coding genes even when spanning several genomic fragments. This sets the stage for a better understanding of the evolutionary history within particular gene families which possess a large number of paralogs and in which frequent gene duplication events occurred.

**Electronic supplementary material:**

The online version of this article (doi:10.1186/s13015-016-0063-y) contains supplementary material, which is available to authorized users.

## Background

Accurate multiple sequence alignments are required as input for a wide variety of different computational analysis techniques in phylogenetics, molecular evolution and comparative genomics. In this contribution we will primarily be concerned with protein coding regions. Tests for inter-residue co-evolution [1] and correlation of conservation with protein structure [2] allow for identification of functional motifs and elements. Protein interfaces and interaction partners can be predicted considering inter-protein co-evolution [1]. These approaches can be used to improve protein structure prediction. Sequence alignments also form the basis for evaluating changes in positive or purifying selection pressures [3] over evolutionary time scales.

Many large protein families, such as transcription factors, growth factors, proteins involved in signaling pathways and membrane proteins, include paralogous members that share highly similar sequence elements. Detailed phylogenies of these protein families — usually referred to as gene trees — are utilized to reveal rapid gene loss and pseudogenization, frequent gene duplication and abundant gene conversion events [4]. The reconstruction of accurate gene trees for protein families, however, has turned out to be one of the most recalcitrant problems in computational biology. This has of course multiple causes. One key issue, which is the main motivation for this contribution, is the availability and quality of the input sequence data.

The protein sequences used to reconstruct large-scale gene families are usually extracted from public protein databases (such as Swiss-Prot [5], NCBI-RefSeq [6]) or from genome annotations (in particular Ensembl [7] or the NCBI genome database [6]). Despite the best efforts of the biocurators’ community and continuing improvements, these data sources contain high levels of errors and inaccuracies [8] that are virtually unavoidable given the volume of data that must be processed to create them. The annotation of a newly sequenced genome is typically achieved by combining *ab initio* and similarity-based gene prediction methods such as those employed in the NCBI eukaryotic genome annotation-pipeline [9]. The first group of *ab initio* gene prediction tools relies on Markov chain models and position-weight matrices for construction of gene models (e.g., geneid [10]). The second class is trained on complementary DNA (cDNA) or RNA sequencing (RNA-Seq) data available for the species of interest to obtain probabilities to build (generalized) hidden Markov Models (AUGUSTUS [11], GENSCAN [12]). Similarity-based methods benefit from available cDNA, expressed sequence tag (EST) or protein data from the same species (producing a *cis*-alignment) or from a closely related species (producing a *trans*-alignment) [13]. Key similarity-based methods in this context are spliced alignment algorithms; these align proteins or cDNA/EST data to a short genomic locus (ProSplign [9], Prot_map [14], GeneWise [15]) or to the whole genome (exonerate -m est2genome [16], GenomeThreader [17]) while allowing for insertions in the target sequence (corresponding to introns) and considering splice-site patterns.

Although many steps within various annotation pipelines have been optimized, some even for decades, they still may make mistakes such as over- and under-predicting small introns and exons. Even extensive EST or RNA-Seq data sets may be incomplete. Both false positive and false negative predictions are propagated by the comparative algorithms and can only be rectified, in part, by the diligent work of human curators.

One particular difficulty is that most available genomes are not finished, i.e., the corresponding genome assemblies consist of many, often short contigs and scaffolds, and genes span over more than one of these genomic units. Although genome quality is improving and long-read techniques [18] are becoming available to a broader community, the issue is likely to persist in the near future. Within the Genbank database, 31.7 % of all eukaryotic genomes and 11.8 % of the animal genomes are at present assembled only to contig-level [19] and even many of the genomes assembled to chromosomes still contain highly fragmented parts.

Standard gene prediction tools and pipelines usually have difficulties with fragmented assemblies. The Ensembl pipeline, for instance, rejects matches covering less than 25 % of the query protein [20]. In the SGP2 framework [21], *ab initio* gene prediction (geneid) and similarity search (tblastx) are combined. SGP2 assumes that hits on different fragments originate from a non-assembled shotgun genome. SGP2 will summarize these hits to one gene prediction by re-scoring of the high-scoring segment pairs. Thus, different, highly similar paralogs tend to be merged into a single gene prediction. The combined mapping/alignment tool GMAP [22], which was originally intended to uncover chimeric ESTs, maps cDNA/ESTs to multiple genomic loci. This method theoretically allows for annotation of genes in a fragmented genome, although to our knowledge application of GMAP has been limited to *cis*-alignments [22]. The Scipio system [23] was developed originally for *cis*-alignments of proteins and cDNAs and later has been extended to *trans*-alignments [24]. It proceeds stepwise: (1) blat alignment, (2) gap closing in the query sequence using a Needleman-Wunsch alignment, (3) assembling of the blat hits, and (4) intron border refinement [24]. Recent refinements to accommodate the needs of particular query genes are described in [25]. The problem of assembling genes from multiple genomic fragments becomes particularly difficult in cases where multiple close paralogs are present. A frequent error is the construction of chimeric gene models that thread through fragments belonging to different paralogs, see e.g., [26]. We addressed this particular issue here by describing an algorithm that identifies the optimal assignment of coding exons to genomic fragments. In contrast to existing methods, which find, separately for each query paralog, the best match(es) in the genome, we solve here a formal assignment problem that identifies the collectively best match of an entire group of paralogous genes to a set of genomic loci.

The naming of genes in public resources adds another level of complication, and another potential source of error for the user, as nomenclature conventions are restricted to individual species or small groups of species. The HUGO Gene Nomenclature Committee is working to establish a coherent naming scheme for the genes in vertebrate genomes, aiming at a nomenclature that actually reflects homology as much as possible [27]. In practice the retrieval of family members relies either on using databases of homologs such as Ensembl Compara [28] and HomoloGene [6], or on the use of similarity-based sequence search tools such as blast [29]. The use of public homology databases unavoidably is limited to the data included by its curators and restricted to the data sources, i.e., genome annotations, that have been selected for inclusion. Recently completed, still poorly annotated genomes are often not yet included.

A detailed exploration of gene families can improve existing annotations and known homologies as retrieved from public data resources. It is unavoidable in practice to expend substantial efforts into data curation to complete that data set with respect to missing genes and to correct individual gene models. Here we present a tool that is designed to assist in the initial data curation step. Specifically, we aim to help the user to improve gene models regarding exon-intron structure, to compile complete sequences from poor, fragmented assemblies and to avoid ambiguities in paralog group assignments.

## Methods

### Pipeline overview

**Fig. 1:**
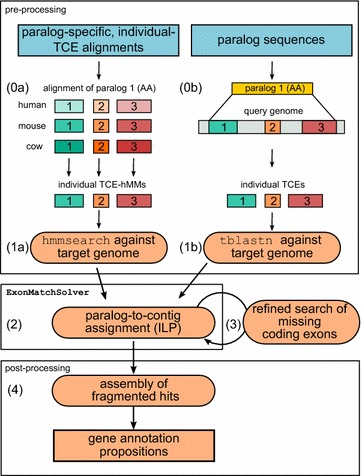
The EMS-pipeline explicitly solves the paralog-to-contig assignment problem. Sequence-matches to individual TCEs are collected in a step-wise procedure applying either tblastn (from single sequences of individual TCEs) or hmmsearch (starting from a sequence alignment for each TCE). Depending on the input, pre-processing steps (0a) or (0b) are performed before similarity search. The *colored boxes* represent TCEs. The pre-processing steps, which are performed separately for all individual TCEs of all paralogs, are exemplified here for one paralog encoded by three exons. For a detailed description of the individual steps, we refer to the "Methods" section. *AA* amino acid sequence, *hMMs* hidden Markov Models, *ILP* integer linear programming problem, *TCE* translated coding exon

The conceptual translation of the coding portion of an individual exon, a translated coding exon (TCE) for short, is treated as elementary building block. To account for gain and loss of exons we envision a hypothetical “ancestor” as an ordered list of TCEs from which each of the observed extant protein sequences can be derived by deletion of TCEs. An exon in an extant sequence therefore may be represented by two or more TCEs. This is the case when a homologous sequence in the same or another species is interrupted by one or more introns. The maximal number of paralogs to be identified in the target genome is either derived from the input or can be specified by the user. The ExonMatchSolver-pipeline (EMS-pipeline) implements a work-flow comprising four main steps: (1) the search of protein sequences or protein-models specific for paralogs and individual TCEs against a complete target genome, (2) the paralog-to-contig assignment formulated as an Integer Linear Programming (ILP) problem, (3) a refined search for exons missing after step 2 relative to the input gene models, and (4) the assembly of fragmented hits and the proposition of gene annotations. The formulation of the ILP is the core of the EMS-pipeline and will be referred to as ExonMatchSolver in the following. The EMS-pipeline produces both a predicted protein sequence for each paralog, and an assignment of each predicted paralog to a paralogous group. The EMS-pipeline accommodates several types of input (see subsection "Implementation and usage"). If paralog-specific and individual-TCE alignment-files are provided, hidden Markov Models (hMM) are built (0a) and used as queries. Otherwise, homologous TCE groups across paralogs within the query genome can be identified in an additional pre-processing step (0b). The overall organization of the underlying workflow is summarized in Fig. 1. A detailed schematic is provided as Additional file 1.

### Exon assembly as an assignment problem

**Fig. 2:**
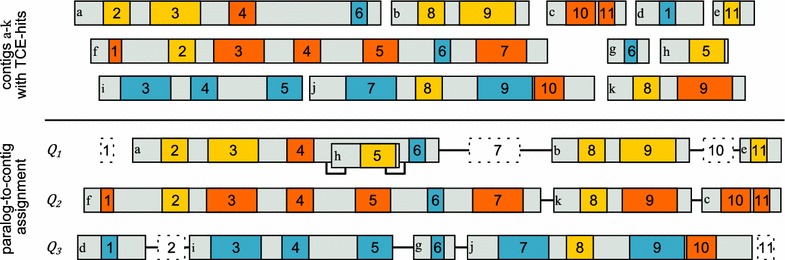
Illustration of the paralog-to-contig assignment problem. In this hypothetical example, each of the three paralogous genes has 11 coding exons which are homologous to the respective exon of the other paralogs (numbers 1–11). The paralogs are distributed over 11 contigs of different sizes, which are denoted by *letters*. TCE-hits on the 11 contigs are *colored* according to the query paralog $Q_i$ that scored best (*yellow* query paralog $Q_1$, *red* query paralog $Q_2$, *blue* query paralog $Q_3$). The *lower* part of the figure shows the assignment identified by the EMS-pipeline. Note, that exon 5 of paralog 1 is inserted into contig a that carries exons 2–4 and 6. Putative missing (or deleted) exons are shown as *dotted boxes*. *TCE* translated coding exon

The key difficulty is the creation of a complete and accurate gene model of the coding sequence on fragmented genome assemblies. Our starting point is a set $\{Q_1,\dots ,Q_N\}$ of *N* paralogous query proteins. For each query protein $Q_j$ we are given a decomposition into its TCEs $(q^j_1,q^j_2,\dots ,q^j_{m_j})$. Furthermore, we are given a set $\{X_1,X_2,\dots , X_n\}$ of contigs and a similarity score $\theta _{ijk}$ measuring how well TCE $q^j_k$ of paralog *j* matches to contig *i*. Figure 2 illustrates the problem setup.

We use the term contig here to refer to a genomic locus harboring at most one gene of interest. If the contigs in the genome assembly are very long, they may have to be subdivided so that each target sequence contains only a single locus of interest e.g., by creation of a new, artificial contig that was not contained in the original assembly. Furthermore, all contigs without significant matches are removed before solving the paralog-to-contig assignment problem.

The assumption that each TCE can be derived from a hypothetical “ancestor” by deletion of TCEs covers all gene families in which the gene structure was not subject to permutations of exons. For instance, if an exon was split in one lineage by insertion of an extra intron, this extra intron boundary can be traced back to the “ancestor” and inserted within all its descendants. TCEs then have to be artificially split at this boundary. After this preparatory step (which is left to the user in the current implementation), the TCE blocks (in the following simply called TCEs for brevity) are numbered consistently, in the sense that homologous TCEs have the same number and $m_j=m$ becomes independent of the paralog. Missing (deleted) TCEs simply remain unmatched.

The quality of a match between query TCE $q^j_k$ to a genomic match *i* in contig $X_i$ is measured by the bitscore $\theta _{ijk}$ computed by either tblastn or hmmsearch [30, 31]. To remove spurious hits, we first employ an *E* value filter. Secondly, TCE-hits that are found alone on one contig without any accompanying hits are subjected to a length-normalization and a bitscore-filtering. For undesirable assignments, we set $\theta _{ijk}=0$.

The paralog-to-contig assignment problem is a combination of a matching problem [32] and an assignment problem [33]. It can be phrased formally as follows:

***Paralog-to-contig assignment problem*** (PCAP)

- **Instance:** A set *Q* of *n* queries (“paralogs”), each of which comprises a non-empty list of TCEs denoted (*j*, *k*) with $1\le j\le n$ and $1\le k\le m_j$; a set *T* of *N* targets (“contigs”), each comprising a list of sites (*i*, *h*) with $1\le i\le N$ and $1\le h\le M_i$; scores $\sigma _{i,h;j,k}$ measuring the similarity of query TCE (*j*, *k*) with target site (*i*, *h*).
- **Solution:** A bipartite matching $\mathcal {M}$ of query TCEs (*j*, *k*) and target sites (*i*, *h*) so that
  1. each target *i* is assigned to at most one query *j*, i.e., $(j,k):(i,h)\in M$ and $(j',k'):(i,h')\in M$ implies $j'=j$, and
  2. if $(j,k):(i,h)\in M$ then there is $(j,k'):(i,h')\in M$ for every TCE $k'$ of the query *j* for which there is a site $h'$ on the same target *i* with $\sigma _{i,h';j,k'}>0$.
  The target sites are interpreted as (parts of) exons so that in instances of practical interest to us, each TCE and each site can be assigned a type $\tau$ so that $\sigma _{i,h;j,k}>0$ if and only if $\tau (i,h)=\tau (j,k)$.
  **Objective function:**
  1 $$\begin{aligned} f(\mathcal {M}) = \sum _{(j,k):(i,h)\in \mathcal {M} } \sigma _{i,h;j,k} \rightarrow \max ! \end{aligned}$$

Since we assume for practical applications to biological data, that each exon type appears at most once on each target *i*, we can suppress the index *h* and set $\theta _{ijk}:=\sigma _{i,h;j,k}$ if there is (*i*, *h*) with $\tau (i,h)=\tau (j,k)$ and $\theta _{ijk}:=-\infty$ otherwise.

We first show that PCAP is a difficult combinatorial optimization problem:

#### **Theorem 1**

*The decision problem version ofPCAP is NP-complete.*

#### *Proof*

We prove the NP-completeness of PCAP by reduction from the ***graph 3-coloring*** problem, which is known to be NP-complete [34].

Consider an arbitrary graph $G=(V,E)$ and an associated PCAP with $n=3$ queries and $m=|E|$ TCEs on each query. For each $i \in V$ we create one target, with $M_i = |\left\{ {i': [i,i'] \in E}\right\} |$ sites. We assign a “type” $\tau \in \mathbb {N}$ to each query TCE and target site, and set $\sigma _{i,h;j,k}=1$ if and only if $\tau (i,h)=\tau (j,k)$ and $\sigma _{i,h;j,k}=-\infty$ otherwise, i.e., query TCEs can only match with target sites of the same type. We assume that there are |*E*| distinct types, each associated with a single edge in *G*. A target *i* contains a site of type $\tau$, if and only if the respective vertex is incident to the corresponding edge. Two targets *i* and $i'$ therefore share a site of the same type if and only if $[i,i'] \in E$. The three queries are constructed as identical lists, each containing TCEs of all |*E*| types. Therefore, any independent set of targets matches to each query, while no query can match two adjacent targets. A solution of the PCAP constructed in this manner, in which every target is assigned to one of the three queries, implies a 3-coloring of *G*. Conversely, if a 3-coloring of *G* exists, it provides a solution of the PCAP.

Finally, it is easy to verify that the PCAP constructed from *G* has polynomial size: There are |*V*| targets, each of which has not more than |*E*| edges, i.e., there are not more than $|V|\,|E|$ target sites and exactly 3|*E*| query TCEs, i.e., the size of the underlying matching problem lives on a graph with $O(|V|^3)$ vertices.

Thus PCAP cannot be easier than graph 3-coloring, which is NP-complete. $\square$

Since Theorem 1 precludes the existence of an efficient solution (unless P=NP), we solve PCAP by means of Integer Linear Programming (ILP). To this end, we have to convert the formal specification of PCAP above into a set of linear constraints. We use the simplified notation for the similarity scoring in terms of $\theta _{ijk}$.

### Solving the paralog-to-contig assignment problem

To formulate the PCAP as an ILP, we consider the binary variables $C_{ij}$ with $C_{ij}=1$, if and only if paralog $Q_j$ is assigned to contig $X_i$, and $C_{ij}=0$ otherwise. Additionally, we introduce the binary variables $E_{ijk}$, with $E_{ijk}=1$, if and only if TCE $q^j_k$ from paralog $Q_j$ is assigned to contig $X_i$, and $E_{ijk}=0$ otherwise. While the variables $C_{ij}$ represent the associations between paralogs and contigs, $E_{ijk}$ represent the associations between the TCEs (of a certain paralog) and the contigs. We then look for an assignment that maximizes the total similarity score:

2 $$\begin{aligned} \max \sum _{i=1}^n \sum _{j=1}^N \sum _{k=1}^{m} \mu _{ij} \theta _{ijk} E_{ijk} \end{aligned}$$

with $$\theta _{ijk}$$ being the bitscore of the respective hit, and $$\mu _{ij}=|\{k | \exists j' : \theta _{ij'k}>0\}|$$ being the number of (groups of homologous) TCE-hits found on contig $$X_i$$, i.e., those where for at least one paralog $$Q_{j'}$$$$\theta _{ij'k}>0$$. In addition to $$\theta _{ijk}$$, which favors matches with a high similarity score, we introduced the factor $$\mu _{ij}$$ to prefer assignments with multiple TCE-hits found on the same contig.

The assignment is subjected to a series of constraints. First, each TCE $q^j_k$ is assigned at most once, and the same contig $X_i$ does not carry more than one paralog $Q_j$.

3 $$\begin{aligned} \forall j,k:\ \sum _{i=1}^n E_{ijk} \le 1 \quad \text {and}\quad \forall i:\ \sum _{j=1}^N C_{ij} \le 1 \end{aligned}$$

Second, a contig $X_i$ is not assigned to paralog $Q_j$, if no TCE-hit $q^j_k$ from paralog $Q_j$ was found on this contig.

4 $$\begin{aligned} \forall i,j \text { s.t.} \not \exists k | \theta _{ijk}>0:\ C_{ij}=0 \end{aligned}$$

Third, contig $X_i$ is assigned to paralog $Q_j$, if and only if at least one TCE $q^j_k$ is assigned to that contig, i.e., $C_{ij}=1$ if and only if $\exists k$ s.t. $E_{ijk} = 1$.

5 $$\begin{aligned} \forall i,j:\ \sum _{k=1}^{m} E_{ijk} - C_{ij} \ge 0\end{aligned}$$

6 $$\begin{aligned} \forall i,j:\ \sum _{k=1}^{m} E_{ijk} - m C_{ij} \le 0 \end{aligned}$$

with *m* being the number of groups of homologous TCEs. Finally, if contig $$X_i$$ is assigned to paralog $$Q_j$$, then all respective TCEs, which are found on this contig, are assigned to it, i.e., if $$C_{ij}=1$$ then $$\forall k$$ s.t. $$\exists j'$$ for which $$\theta _{ij'k}>0$$, it holds that $$E_{ijk}=1$$. Otherwise, if $$\forall j'$$$$\theta _{ij'k} \le 0$$, then $$E_{ijk}=0$$.

7 $$\begin{aligned} \forall i,j :\ \mu _{ij} C_{ij} - \sum _{k | \theta _{ijk} > 0} E_{ijk} \le 0 \end{aligned}$$

8 $$\begin{aligned} \sum _{i=1}^n \sum _{j=1}^N \sum _{k | \forall j' : \theta _{ij'k} \le 0} E_{ijk} = 0 \end{aligned}$$

This simple ILP determines an optimal assignment $C_{ij}$ of paralog $Q_j$ to contig $X_i$, which can now be used to determine the sequences of paralogs. In these gene models, however, there still may be small or divergent exons missing, for which no significant hits were obtained.

### Post-processing

To alleviate this limitation of the initial similarity search, two additional search steps are preformed: (1) Local tblastn searches limited to only those contigs, where hits were identified for at least one TCE-model may identify additional candidate TCEs, (2) Spliced alignments of the query sequence on un-assembled contigs are used to increase the sensitivity. In contrast to local tblastn and hmmsearch, spliced alignment tools such as ProSplign align the full-length protein query sequence to a genomic sequence fragment. This makes it possible to detect short TCEs that do not yield significant scores in genome-wide searches.

Upon compiling the final gene models, three cases appear. (i) In the simplest and ideal case, a paralog is located on a single contig with all TCEs fully covered and identified. No other assembly steps are required. (ii) The paralog is distributed over multiple contigs such that every contig contains a sequence of consecutive TCE-hits in the correct order. In this case, the different fragments can be concatenated unambiguously, accounting for the TCE order and the strandedness of the fragments. (iii) The TCE-hits identified on a contig are ordered correctly but they are not consecutive. For example, $X_1$ might carry TCEs *p* ... *q* and *r* ... *s*, but $q+1 ... r-1$ are located on $X_2$. This occurs if the genome assembly is erroneous or if the two “contigs” are actually (pieces of) two scaffolds that interleave (e.g., Fig. 2, contigs a and h). To account for these cases, we attempt to insert $X_2$ in the appropriate place of $X_1$. The hypothesis of how two or more contigs have to be interleaved is entirely determined by the order of the exons on the query gene, and is therefore unique. If the contig contains stretches of Ns (indicating missing sequence at the scaffold level), the contig parts are interleaved there. Otherwise, the sequence is inserted at an arbitrary locus for the preservation of the correct exon order. A spliced alignment tool is then run again on the merged contigs to refine the gene model.

### Implementation and usage

The EMS-pipeline can be run in three general modes depending on the information available as input (see Additional file 1). As the minimal input, the protein sequences of all paralogs of interest from a well annotated species and the complete target genome must be provided as fasta files. In “fasta-mode”, homologous TCE groups are identified by a tblastn of the query protein against the query genome (Fig. 1, step 0b). To reduce false assignments of TCEs to homologous groups, we compute a background distribution of pairwise similarity scores from the matches of a query TCE against all other TCEs of the same paralog. This information is used to determine a cut-off value $\hat{\theta }_{j}$ corresponding to a user-defined *z*-score to remove likely promiscuous matches between non-homologous TCEs. In order to further reduce the false assignments of short TCEs to homologous groups of putative lengthy TCEs, TCEs with lengths below a length cutoff are excluded. This step may require manual inspection if the analysis shows that originally annotated exons are split in some of the family members. Then the assignment of TCEs given as input to step 1 may need to be adjusted.

The “alignment-mode” can be used when the exon-intron structure of the paralogs is already known and the user has access to well-annotated sequences from several species. Input protein alignments are converted to hMMs applying the HMMER3 suite [31] and are then used to scan the conceptually translated target genome (Fig. 1, step 0a). This improves both specificity and sensitivity of the tool. It can be used iteratively to improve results from a first set of searches starting from a single query.

Alternatively, the user can provide information on TCE-homology of the query protein sequences in “custom-mode” to include as many homologous TCE groups as possible.

Exact exon-intron structure of the query sequences in the target genome and in the query genome, if necessary, are inferred by means of a spliced alignment tool, by default ProSplign [9]. Alternatively, exonerate [16] can be used, which is faster but less sensitive [24]. In cases in which very long introns are predicted, the EMS-pipeline switches to exonerate automatically.

The ILP solver can be used to obtain alternative, sub-optimal assignments. This is particularly useful to judge the reliability of the solution.

After completion of the first assignment by the ExonMatchSolver, the TCE-search is refined by running hmmsearch and tblastn with more sensitive settings as described above. The majority of TCE-hits for one paralog is usually assigned to one contig. A spliced alignment tool is used to align the query sequences to these contigs. The list of hits is augmented with these hits and the final paralog-to-contig assignment is computed.

Different contigs assigned to the same paralog are then merged/assembled. In some cases, contigs are interleaved. If so, the sequence of a single coding exon is inserted into the genomic area between the closest TCE-hits on the main fragment. If this region contains stretches of three or more consecutive Ns, the sequence is inserted in one of these regions. Large blocks of Ns are substituted by the insert-sequence. If the contig has no N-blocks in the appropriate region, the coding exon is inserted together with flanking Ns. The resulting edited “scaffolds” are again compared against the query sequences via a spliced alignment.

The resulting protein models as well as the input protein sequences are finally turned over to the Scipio gene annotation pipeline. Gene annotation proposed by tblastn, exonerate, ProSplign as well as Scipio should be compared by the user to infer paralog assignment and gene structure. The assignment list created by the ExonMatchSolver and the list of any remaining, questionable, single coding exons is available for manual evaluation.

### Assessment of the ExonMatchSolver’s performance by simulations

In order to estimate performance and running time of the core step, we tested the ExonMatchSolver on simulated data. Protein sequence evolution is simulated with ALF [35] for two hypothetical species (query and target) allowing for insertions, deletions, substitutions and duplications in a randomly generated protein sequence (branch length, $n= 50$, indel-rate = 0.0005, standard settings otherwise). This first step implements the evolution of one ancestor protein sequence to a fixed number of paralogs with an average of 2.5 % indels per sequence. The simulated protein sequences are divided into homologous pieces according to exon lengths sampled from a data set of human protein coding genes originating from Ensembl (Lozada-Chavéz I., in preparation). These exons are simulated to evolve independently (branch length, $n=20$, about 1 % indels per sequence) without allowing for duplications in a second step representing recent evolutionary changes. Exons of the single paralogs are distributed to different units (representing genomic fragments) with varying fragmentation levels. The fragmentation level is calculated as the average number of exons per fragment. Scoring of the query protein or TCEs against the target TCEs is performed with blastp (*E* value $<$ 0.0001).

Performance of the ExonMatchSolver is assessed in comparison with a “greedy” method. We consider the assignment of a paralog to a unit greedy, if it is solely determined by the identity of the unit which retrieved the best bitscore with the respective full length query paralog. Accuracy and running time of the ExonMatchSolver and the greedy method both depend on the individual random protein sequences that were simulated as well as on the exon sizes that are sampled from the exon length data set. To be able to directly compare these results, estimation of accuracy and running time are performed on the same set of simulated protein sequences. For the accuracy estimation, fragmentation is repeated 1000 times for each fragmentation level with a fixed number of paralogs (8) and exons (12). The running time of the ExonMatchSolver is estimated for different numbers of exons and paralogs and a fixed fragmentation level (7.7 exons per fragment on average). The estimated user time is averaged for 20 different fragmentations on the same simulated data. Resident Set Size (rss) is used as an estimate of memory.

## Results

### Performance on simulated data

**Fig. 3:**
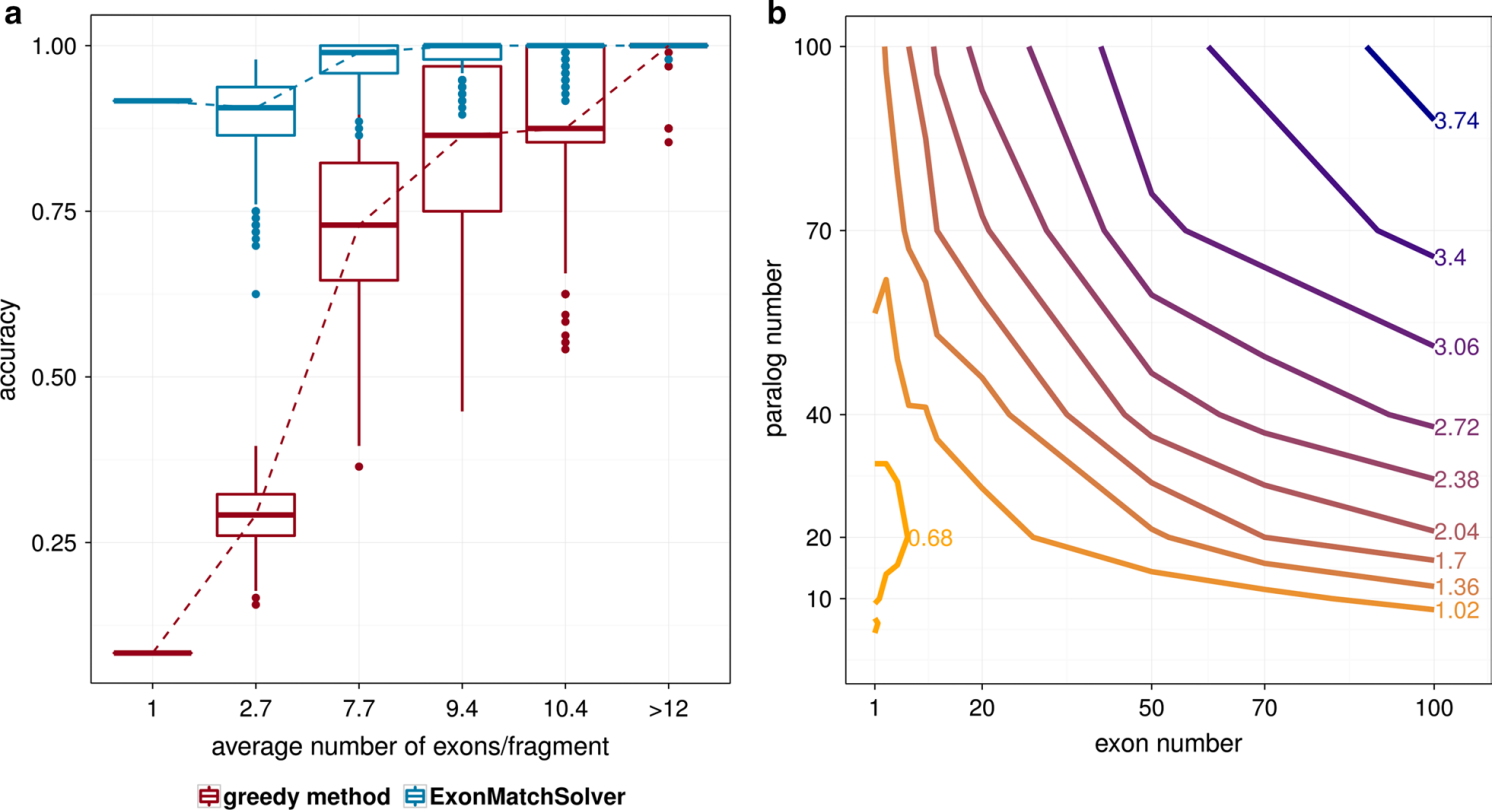
Accuracy and running time of the ExonMatchSolver on simulated data. **a** Dependence of the accuracy on the fragmentation level in comparison with a greedy approach. Eight paralogs, each possessing 12 exons, were simulated in two species using ALF with branch length $n=50$ for generation of paralogs and branch length $n=20$ for evolution of single exons. Fragmentation of exons across units was simulated 1000 times for each fragmentation level. **b** Dependence of the running time on paralog and exon number. *Color* changes of contour lines from *yellow to dark blue* indicate an increase in running time. *Contour lines* are labeled with the $\log _{10}$ of the running time. Different numbers of paralogs (4, 6, 8, 10, 20, 40, 70, 100) and exons (1, 3, 5, 7, 10, 12, 20, 50, 70, 100) were simulated using ALF with parameters specified in the ”Methods” section and 7.7 exons per fragment on average. Running time was estimated as the mean of the user time of 20 runs with different fragmentation levels of the same simulated sequence data

Accuracy of the ExonMatchSolver was estimated on simulated data and compared to the greedy method’s accuracy on the same data set. For the simulated sequences of eight paralogs with 12 exons, the ExonMatchSolver solved the paralog-to-contig assignment more accurately than the greedy method if paralogs were fragmented across several units. Accuracy of the ExonMatchSolver was as good as that of the greedy method for non-fragmented paralogs (Fig. 3a). As expected, accuracy of both methods decreased with higher fragmentation of the genome, indicated by a lower number of exons per fragment. While the accuracy of the greedy method dropped by more than 90 % from 1 to 0.08, the accuracy of the ExonMatchSolver solution did not fall below 0.91 even for the highest fragmentation levels. Thus, the ExonMatchSolver clearly outperformed the greedy method in assignment of paralogs to the correct units, which equalize contigs in non-simulated data.

In some simulations, the maximal accuracy of the ExonMatchSolver might be slightly lower than the accuracy of the greedy method at high fragmentation levels. This can be attributed to false negative hits representing short or very divergent exons that are not retrieved by the ExonMatchSolver. In the greedy comparison such false negatives do not occur because there, contigs are queried with the full-length protein. Although such false negative hits are in part retrieved in the post-processing step of the EMS-pipeline (as seen for the show case examples below), this step was not included in the performance tests for the ExonMatchSolver.

The running time of the ExonMatchSolver was in the range of a few seconds to minutes in dependence on the number of exons and paralogs (Fig. 3b, see Additional file 1). Instances with 100 exons and 100 paralogs, the largest number of exons and paralogs tested, were an exception to this rule as they required about 2.5 h of running time and 228 GB of memory on average (see Additional file 1). For more moderate numbers of 70 exons and up to 20 paralogs, running time was below one minute while at most 3.5 GB of memory was required. The running time and memory increased to more than 15 min and 35.4 GB, respectively, when exceeding 50 exons and 70 paralogs. The ExonMatchSolver thus solved even instances with extremely high numbers of paralogs and exons in feasible time. For most biologically relevant instances, memory requirements do not exceed the resources provided by a contemporary notebook.

### Performance on real data - two showcase examples

We selected two difficult examples, latrophilin receptors and arrestins to demonstrate the usefulness of the full EMS-pipeline on real data. Small differences in the exon-intron structure of the input paralogs are handled as if all paralogs derive from an ancestor that contains all coding exons.

#### Arrestins

**Fig. 4:**
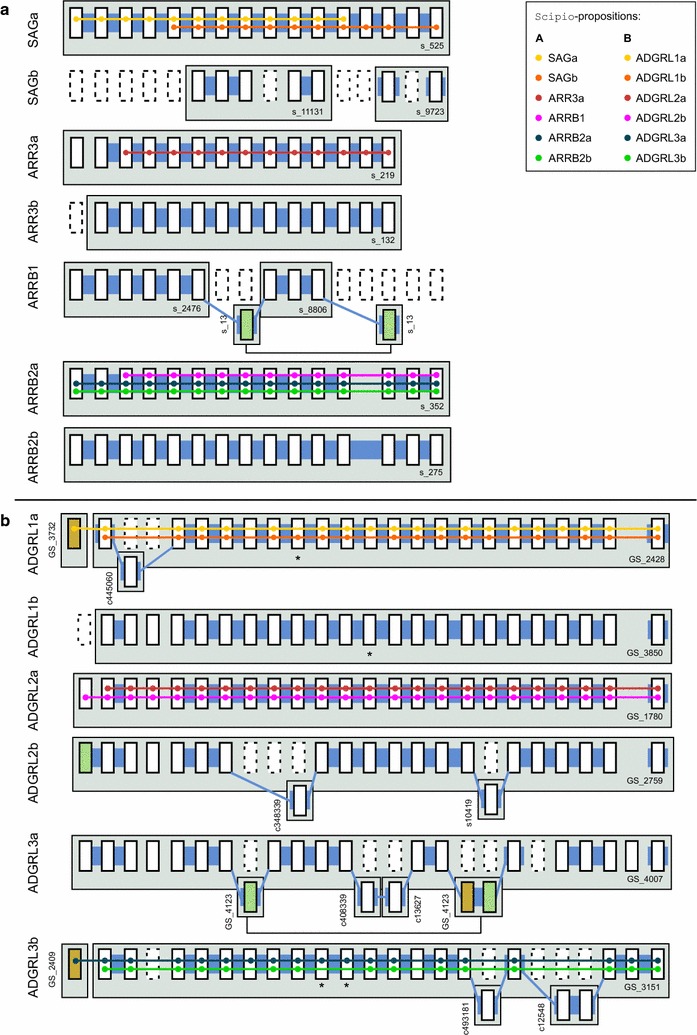
Illustration of the paralog-to-contig assignment for arrestin paralogs in pufferfish (**a**) and latrophilin paralogs in cod (**b**). All known zebrafish paralogs of the respective families were used as queries. Homologous coding exons that were detected by the EMS-pipeline and Scipio, resp., are shown as *black open boxes* on their respective contigs (*grey boxes*). Putatively missing (or deleted) coding exons are denoted by *dotted boxes*. False positive TCE-hits that were included in the ExonMatchSolver-solution, but not annotated by the spliced alignment tool are indicated by *light green boxes*. False positives appearing in the final output of either tool are indicated by *brown boxes*. The solution of the EMS-pipeline considering all of its stages is highlighted by *broad blue paths* in the back of the exons. Scipio’s best scoring proposition for each query is illustrated as *colored dots* and *paths*. In addition, true positive TCEs that were detected by the ExonMatchSolver, but not by the spliced alignment tool, are marked with an *asterisk* (*). Note that Scipio with cross-species default options did not propose any hit for ARR3b. *GS* genescaffold, *c* contig, *s* scaffold, *TCE* translated coding exon

Arrestins are signaling and scaffolding molecules best known for their interaction with G-protein Coupled Receptors (GPCRs). Four paralogs are encoded by 15–16 exons in humans (*Homo sapiens*), SAG, ARRB1, ARRB2 and ARR3. All arrestin genes except ARRB1 are duplicated in zebrafish as a result of the fish-specific whole genome duplication (FSGD) event [36]. The genes span a length of up to 82 kbp. Overall, the exon-intron structure is conserved except for two intron losses in zebrafish ARRB2b and ARR3a. There are two micro-exons, exons 1 and 15, with less than 15 nucleotides (nt) in length. These are particularly challenging to infer. We aimed to predict the seven arrestin paralogs in pufferfish (*Takifugu rubripes*, Ensembl*FUGU 4.0*) with the EMS-pipeline in “custom-mode” starting from protein sequences in zebrafish. If no experimentally verified entries were available in genbank (NP_001153294.1, AAH76177.1, AAI52656.1, NP_957418.1), the annotations were extracted from Ensembl, *Zv9*. The last exon of SAGb was identified by an additional tblastn-search with SAGa as query. In the following, values for the number of contigs, to which paralogs were assigned, refer to the final output of the EMS-pipeline after spliced alignment of the assembled loci. TCEs were considered as found even if they were only partially identified. In the same sense, extensions of TCEs by the spliced alignment tools and additional alignment hits on the same fragment were not considered as false positives. For the arrestins, the EMS-pipeline identified all expected seven arrestin paralogs situated on nine different contigs (see Fig. 4a and Additional file 1). Five paralogs were (nearly) completely encoded on one contig each, while only parts of the other two, SAGb and ARRB1, were sequenced. SAGb and ARRB1 were fragmented covering two genomic units each.

For comparison, we ran Scipio, which identified four different arrestin loci with cross-species default options suggesting the loss of three paralogs relative to zebrafish. Considering the best scoring results for each query, Scipio assigned two different arrestin paralogs to *scaffold_525*, while three other paralogs were assigned to *scaffold_352*. No hits were suggested for ARR3b. Running Scipio with optimized options for arrestin genes allowed for an increased assembly size and increased sensitivity for detection of small exons. Therefore, seven different loci were proposed among the alternative results (see Additional file 1 for a phylogenetic tree of all alternative Scipio annotations). This is in accordance with the results proposed by the EMS-pipeline. Four out of the seven paralogs were correctly identified by Scipio, while the other three matched loci already assigned to a paralogous group. In other words, none of the contigs harboring SAGb, ARRB1, and ARRB2b appeared as a best scoring result in the Scipio predictions. In this example, the EMS-pipeline with ProSplign as spliced alignment component correctly identified two coding exons that remained undetected by Scipio. The short coding exon 1 of ARR3a (eight nt) could be annotated manually with the help of a local blastn search using the nucleotide sequence of the corresponding zebrafish exon as query. It was missed by both the EMS/ProSplign- pipeline and by Scipio.

#### Latrophilins

The latrophilins (ADGRL1, ADGRL2 and ADGRL3) belong to the family of adhesion GPCRs and are encoded by 22–26 exons spanning a total length of up to 210 kbp in zebrafish [37]. A recent phylogenetic study proposed the duplication of ADGRL1 and ADGRL2 in zebrafish resulting in a greatly shortened N-terminus [38]. The five paralogous family members have a highly similar exon-intron structure in zebrafish thus fitting well with the application scenario of the EMS-pipeline. In ADGRL1a and ADGRL1b, exon 5 is split into three independent exons in comparison to the other paralogs, resulting in 25 homologous exon groups. We aimed to annotate ADGRL1, ADGRL2 and ADGRL3 and possible additional paralogs in cod (*Gadus morhua*, Ensembl*gadMor1*), which shares the FSGD with zebrafish. As a starting point, we chose the annotation of latrophilin paralogs in the well assembled genome of zebrafish (Ensembl*GPRCz10*). To obtain a trustworthy query, these were manually curated adding small, missing exons identified by tblastn with human latrophilins as queries. During curation, an additional paralog, ADGRL1b, was identified. The starting data set thus comprised six latrophilin paralogs, all of which were also identified in cod with the EMS-pipeline.

Presumably due to missing data, in total, the sequence of five different single TCEs was missing for all latrophilin paralogs in zebrafish in total. As a byproduct, the ExonMatchSolver will keep TCE-hits even if the fragment is scored with a different paralog-model of the same TCE only. This results in detection of coding exons that might either be missing from the query paralog or represent a pseudogenic exon (marked by asterisk, Fig. 4b).

The EMS-pipeline identified all six paralogs existing in zebrafish situated on 14 different fragments in cod. In contrast, Scipio [39] placed the latrophilin paralogs onto five different contigs or scaffolds in cod when run under cross-species default options (see Fig. 4b and also Additional file 1). Considering the best scoring results only, the tool proposed the existence of three different latrophilin loci. At these loci, Scipio proposed each of the recently duplicated paralog-pairs shared the exact same coordinates on one fragment. If instead, the user inspected the alternative results for each paralog, Scipio’s next-best scoring fragments did not necessarily correlate with the correct contigs that were found by the EMS-pipeline. This was the case for exon 1 of ADGRL1a and ADGRL3b, which could be identified as false negative hits by manual inspection. The EMS-pipeline instead suggested eight different contigs to be interleaved with four of the main fragments. Eight of these nine TCE-hits, proposed in the final output, likely represent true exons that were situated on short fragments remaining from an incomplete genome assembly. In the available annotation of cod, no further genes were annotated on these fragments, supporting the correct paralog-to-contig assignment.

The ninth hit corresponds to exon 23 of the gene CELSR1b encoding part of a secretin-like domain thus representing a false positive hit of the EMS-pipeline. Exons 15–20 of the latrophilin genes code for this domain, common to the whole class of adhesion GPCRs. Inspection of the initial tblastn-hitlist retrieved several high scoring hits of more distant paralogs (e.g., ADGRL4, ADGRE5, and unnamed genes with GPCR-domains) that all possess this domain.

The use of exonerate as a spliced alignment tool caused the EMS-pipeline to miss the short exon 4 in all latrophilin paralogs (15 nt), the short exon 24 in ADGRL3a (18 nt), and the diverse exon 1 of ADGRL2a that were identified by Scipio in the alternative propositions. We therefore recommend to use ProSplign with the EMS-pipeline whenever sufficient computational resources are available. Furthermore, the results of Scipio that are additionally returned by the EMS-pipeline can provide further improvement but require manual inspection.

Interestingly, in both, cod and zebrafish ADGRL2b and ADGRL1b, the exon-intron structure and overall protein length were conserved relative to ADGRL2a and ADGRL1a. This contradicts the proposed truncation of these two genes reported in [38] and emphasizes the need to manually curate database annotation carefully considering differences in gene structure of paralogous genes.

## Discussion

Applying a decomposition of proteins into translated coding exons and separation of homologs into their paralogous groups allows the EMS-pipeline to build models for individual paralog-specific translated coding exons (TCEs). Combining the strengths of different well-established methods and tools (ProSplign, exonerate, tblastn, HMMER and Scipio) that translate between the level of protein and genomic sequence, and novel algorithmic approaches (the automated paralog-to-contig assignment), the EMS-pipeline provides a comprehensive and flexible toolbox for manual, high-quality curation of gene annotations. The heart of the pipeline is the ILP formulation of the paralog-to-contig assignment problem referred to as ExonMatchSolver, which is NP-complete. The ExonMatchSolver solves the assignment problem within seconds or minutes for most biologically relevant numbers of paralogs and exons in simulations. Even for high numbers of paralogs, which might occur in polyploid species such as the octaploid sugar cane [40], the running time does not exceed one hour for up to 70 exons. However, genes with more than 70 exons are rare for human and most other animals [41].

The EMS-pipeline helps to overcome many of the critical problems arising from highly fragmented draft genome assemblies as demonstrated with simulated data as well as with two real life examples. The only program that has been targeted to solve a similar problem, to our knowledge, is Scipio [23]. As suggested by one reviewer, one could alternatively use a maximum weight bipartite matching to identify the correct assignment of paralogous groups from alternative Scipio solutions or built a phylogenetic tree of all alternative Scipio annotations together with the query paralog sequences. As in our example such phylogenetic trees are not always very straightforward to interpret and may also require extensive manual inspection for identification of the correct paraolg-to-contig assignment (Additional file 1). The situation becomes particularly difficult in cases such as the latrophilin example, where Scipio returned chimeric gene models composed of TCEs from different paralogs. The EMS-pipeline is designed to specifically fill this gap for detailed exploration of the evolution of a specific gene family of interest. The explicit use of exon-intron structures and the exon-centric computation of protein similarities furthermore improves the accuracy of paralog identification.

Given the diverse sources of errors and exceptional cases, we have not attempted to construct a fully automatic pipeline, but rather a tool to assist in manual data curation. As a similarity-based method, it depends heavily on the availability of high quality protein sequences (or alignments) as input queries. Erroneous exon annotations or splice site predictions leading to erroneous translated coding sequences in the input unavoidably will be carried over to the results and cannot easily be identified by automatic means.

At present, there are no databases that simultaneously provide both, paralogy information and accurate information on exon-intron structure. The Exon-Intron database (EID) [42] and SpliceDB [43] do not provide information on paralogs; Ensembl Compara on the other hand, does not provide homology information for individual exons. The lack of a gold standard makes it unfeasible to quantitatively benchmark the EMS-pipeline on real data. Therefore, we demonstrated the superior accuracy of the ExonMatchSolver in comparison with a greedy method on simulated data. On real data, we had to rely on a few difficult use cases for which a detailed manual curation was possible.

In its present state, the EMS-pipeline has several limitations. Most importantly, we assume a largely conserved exon-intron structure of the paralogs, a situation that is very often encountered for vertebrate genes. Nonetheless, the exon-intron structure of distant relatives may differ strongly. Largely distinct gene structures can also be accommodated by treating the respective genes as separate paralogous groups. However, cases of recognizable structural similarity together with changing variability might be difficult to handle. Furthermore, we assume that a fairly complete collection of paralogs is used as an input. The paralog-to-contig assignment step may yield incorrect results if the *a priori* estimate of the number of paralogs is incorrect (as in the latrophilin example). In particular, this may lead to the inclusion of more distant, spurious solutions or result in fragmented gene models. In these cases, a manual inspection of the results thus appears unavoidable. We therefore have designed the EMS-pipeline to streamline and simplify the process of manual post-processing that is required for most fragmented genes.

We plan several improvements in future releases of the EMS-pipeline in response to exceptional cases that we have encountered in practical tests so far: The number of paralogs in a genome can presumably be estimated by a more careful analysis of the spectrum of similarity scores. This should help to largely prevent the inclusion of false positives to “compensate” for lineage-specific gene losses and would be useful also when studying gene families with many levels of paralogy, i.e., large numbers of nested gene duplications. It may also be possible to improve the accuracy of the initial, score-based assignments of coding exons to paralogs by using reciprocal best hit heuristics rather than relying on the bitscores $\theta _{ijk}$ of the query matches alone.

## Conclusion

Besides Scipio [23], the EMS-pipeline is, to our knowledge, the only toolkit that can deal with the fragmentation of genes across different contigs in a systematic manner. Nevertheless, consideration of paralogs that are fragmented across different genomic units and their exon-intron structure is necessary to build high quality gene models for detailed phylogenetic and evolutionary analyses. As shown in the examples above, Scipio has substantial difficulties in distinguishing between close paralogs. A closer inspection of erroneous Scipio predictions indicates that these are often the result of incorrect or missing combinations of gene fragments. It therefore seems to be important to explicitly consider and solve the paralog-to-contig assignment problem instead of just selecting best scoring fragments. In particular in the presence of incomplete data, simple protein-level similarity scores are often insufficient to correctly assign partial or even complete protein sequences to the correct paralogous group. Treating this issue as an assignment problem as realized in the EMS pipeline, largely alleviates this particular difficulty of genome annotation.

## Additional file

10.1186/s13015-016-0063-y Detailed illustration of the EMS-pipeline. Extended schematic on steps, user-options and input-modes of the EMS-pipeline. The starting points for the three different modes of the EMS-pipeline are illustrated by *red*, *yellow* and *green dots* as are the different input files required for the respective mode. User options are given on the left and right side in *yellow*. See legend for further explanation. *hMM* hidden Markov Model, *TCE* translated coding exon, *WGD* whole genome duplication.
10.1186/s13015-016-0063-y Phylogenetic tree of arrestin annotations from Scipio. Phylogenetic tree of arrestins as annotated by Scipio in pufferfish together with query orthologs from zebrafish. Scipio was run in sensitive mode (modified options: max_assemble_size = 50000, min_score = 0.1, exhaust_align_size = 50000, region_size = 90000). The alignment and neighbor joining-tree on protein level were built with Clustal Omega 1.2.1 (Sievers et al. 2011 [1]) on all columns of the alignment (A) and on all columns that did not contain any gaps (B). The alternative solutions of Scipio on the same genomic unit may slightly differ in dependence on the query paralog it was retrieved with (indicated by first part of the node label). The zebrafish paralogs are denoted as SAGa,b, ARRB1, ARRB2a,b and ARR3a,b. The solution proposed by the EMS-pipeline is highlighted in green. ARRB1 is situated on scaffold_2476_scaffold_8066. Due to long branch attraction, missing data and sequence divergence, zebrafish and pufferfish orthologs do not always form monophyletic groups. This makes inference of paralog-to-contig assignments based on the phylogenetic tree difficult. The trees were edited and displayed with Dendroscope 2.6.1 (Huson et al. 2007 [2])
10.1186/s13015-016-0063-y Memory and time requirements of the ExonMatchSolver on simulated data. The raw data is provided as Addtional_file3.xlsx. Raw data on memory and time assessment of the ExonMatchSolver on simulated data. The memory and time requirements of the ExonMatchSolver were measured for different numbers of exons (1, 3, 5, 7, 10, 12, 50, 70, 100) and paralogs (4, 6, 8 10, 20. 40, 70, 100) on data simulated with ALF. Memory was estimated as Resident Set Size. Memory and running time were sampled for each combination of paralogs and exons for 20 independent runs of fragmentation and averaged.
10.1186/s13015-016-0063-y Table of paralog-to-contig assignments of arrestins in pufferfish. Performance of Scipio and the EMS-pipeline in prediction of arrestin genes in pufferfish. The pufferfish genome FUGU 4.0 (Ensembl) was queried with zebrafish protein sequences (NP_001153294.1, AAH76177.1, AAI52656.1, NP_957418.1 and annotations from Ensembl Zv9). Scipio was run with “cross-species default options” (min_identity = 60, max_move_exon = 6, blat_score = 15, blat_identity = 54, multiple_results, region_size = 10000, exhaust_align_size = 15000, results given in bold) and in a more sensitive mode (modified options: max_assemble_size = 50000, min_score = 0.1, exhaust_align_size = 50000, region_size = 90000). The sensitive Scipio-mode included all hits of the cross-species default options. If scores deviated, these are separated by “/”. As Scipio was run with the multiple_results-option, several hits are occasionally returned; these are indicated by a number in brackets in the paralog-column. The EMS-pipeline was run in “custom-mode” with ProSplign as spliced alignment tool. TCE-numbering refers to the homologous TCE-groups. Hits were considered even if they were partial only. *fp* false positive, *s* scaffold.
10.1186/s13015-016-0063-y Table of paralog-to-contig assignments of latrophilins in cod. Performance of Scipio and the EMS-pipeline in prediction of latrophilin genes in cod. The cod genome gadMor1 (Ensembl) was queried with zebrafish protein sequences (Ensembl GPRCz10). Scipio was run with “cross-species default options” (min_identity = 60, max_move_exon = 6, blat_score = 15, blat_identity = 54, multiple_results, region_size = 10000, exhaust_align_size = 15000). Scipio occasionally returned several hits; these are indicated by a number in brackets in theparalog-column. The EMS-pipeline was run in “custom-mode” with exonerate as spliced alignment tool. The TCE numbering refers to the homologous TCE-groups. Hits were considered even if they were partial only. *c* contig, *fp* false positive, *GS* GeneScaffold, *s* scaffold.

## References

[1] Juan Dd, Pazos F, Valencia A. Emerging methods in protein co-evolution. Nature Rev Genetics. 2013;14(4):249–61 (2013). doi:10.1038/nrg341. 10.1038/nrg3414 23458856

[2] Celniker G, Nimrod G, Ashkenazy H, Glaser F, Martz E, Mayrose I, Pupko T, Ben-Tal N (2013). ConSurf: Using evolutionary data to raise testable hypotheses about protein function. Israel J. Chem.

[3] Nowick K, Fields C, Gernat T, Caetano-Anolles D, Kholina N, Stubbs L (2011). Gain, loss and divergence in primate zinc-finger genes: A rich resource for evolution of gene regulatory differences between species. PLoS One.

[4] Cortesi F, Musilová Z, Stieb SM, Hart NS, Siebeck UE, Malmstrøm M, Tørresen OK, Jentoft S, Cheney KL, Marshall NJ, Carleton KL, Salzburger W (2015). Ancestral duplications and highly dynamic opsin gene evolution in percomorph fishes. Proc Natl Acad Sci USA.

[5] The UniProt consortium (2015). UniProt: a hub for protein information. Nucleic Acids Res.

[6] Sayers EW, Barrett T, Benson DA, Bolton E, Bryant SH, Canese K, Chetvernin V, Church DM, DiCuccio M, Federhen S, Feolo M, Fingerman IM, Geer LY, Helmberg W, Kapustin Y, Krasnov S, Landsman D, Lipman DJ, Lu Z, Madden TL, Madej T, Maglott DR, Marchler-Bauer A, Miller V, Karsch-Mizrachi I, Ostell J, Panchenko A, Phan L, Pruitt KD, Schuler GD, Sequeira E, Sherry ST, Shumway M, Sirotkin K, Slotta D, Souvorov A, Starchenko G, Tatusova TA, Wagner L, Wang Y, Wilbur WJ, Yaschenko E, Ye J (2012). Database resources of the National Center for Biotechnology Information. Nucleic Acids Res.

[7] Cunningham F, Amode MR, Barrell D, Beal K, Billis K, Brent S, Carvalho-Silva D, Clapham P, Coates G, Fitzgerald S, Gil L, Girón CG, Gordon L, Hourlier T, Hunt SE, Janacek SH, Johnson N, Juettemann T, Kähäri AK, Keenan S, Martin FJ, Maurel T, McLaren W, Murphy DN, Nag R, Overduin B, Parker A, Patricio M, Perry E, Pignatelli M, Riat HS, Sheppard D, Taylor K, Thormann A, Vullo A, Wilder SP, Zadissa A, Aken BL, Birney E, Harrow J, Kinsella R, Muffato M, Ruffier M, Searle, Stephen MJ, Spudich G, Trevanion SJ, Yates A, Zerbino DR, Flicek P (2015). Ensembl 2015. Nucleic Acids Res.

[8] Carugo O, Eisenhaber F. Data Mining Techniques for the Life Sciences. Methods Mol Biol. vol. 609. New York: Humana Press; 2010.

[9] Thibaud-Nissen F, Souvorov, Alexander Murphy, Terence, DiCuccio M, Kitts P. Eukaryotic Genome Annotation Pipeline, Berthesda. 2013. http://www.ncbi.nlm.nih.gov/books/NBK169439/

[10] Guigó R (1998). Assembling genes from predicted exons in linear time with dynamic programming. J Comp Biol.

[11] Stanke M, Waack S (2003). Gene prediction with a hidden Markov model and a new intron submodel. Bioinformatics.

[12] Burge C, Karlin S (1997). Prediction of complete gene structures in human genomic DNA. J Mol Biol.

[13] Brent MR (2008). Steady progress and recent breakthroughs in the accuracy of automated genome annotation. Nature Rev Genetics.

[14] Softberry I. Prot\_map. Softberry, Inc. http://linux1.softberry.com/berry.phtml?topic=prot_map&group=help&subgroup=xmap Accessed 20 Jun 2015.

[15] Birney E (2000). Using GeneWise in the Drosophila annotation experiment. Genome Res.

[16] Slater, Guy St C, Birney E. Automated generation of heuristics for biological sequence comparison. BMC Bioinformatics. 2005;6:31. doi:10.1186/1471-2105-6-3. 10.1186/1471-2105-6-31 PMC553969 15713233

[17] Gremme G, Brendel V, Sparks ME, Kurtz S (2005). Engineering a software tool for gene structure prediction in higher organisms. Inform Software Technol.

[18] Eid J, Fehr A, Gray J, Luong K, Lyle J, Otto G, Peluso P, Rank D, Baybayan P, Bettman B, Bibillo A, Bjornson K, Chaudhuri B, Christians F, Cicero R, Clark S, Dalal R, Dewinter A, Dixon J, Foquet M, Gaertner A, Hardenbol P, Heiner C, Hester K, Holden D, Kearns G, Kong X, Kuse R, Lacroix Y, Lin S, Lundquist P, Ma C, Marks P, Maxham M, Murphy D, Park I, Pham T, Phillips M, Roy J, Sebra R, Shen G, Sorenson J, Tomaney A, Travers K, Trulson M, Vieceli J, Wegener J, Wu D, Yang A, Zaccarin D, Zhao P, Zhong F, Korlach J, Turner S (2009). Real-time DNA sequencing from single polymerase molecules. Science.

[19] NCBI. Genome Report. 2015. ftp://ftp.ncbi.nih.gov/genomes/GENOME\_REPORTS/eukaryotes.txt. Accessed 29 Apirl 2015.

[20] Curwen V, Eyras E, Andrews TD, Clarke L, Mongin E, Searle SMJ, Clamp M (2004). The Ensembl automatic gene annotation system. Genome Res.

[21] Parra G, Agarwal P, Abril JF, Wiehe T, Fickett JW, Guigó R (2003). Comparative gene prediction in human and mouse. Genome Res.

[22] Wu TD, Watanabe CK (2005). GMAP: a genomic mapping and alignment program for mRNA and EST sequences. Bioinformatics.

[23] Keller O, Odronitz F, Stanke M, Kollmar M, Waack S (2008). Scipio: using protein sequences to determine the precise exon/intron structures of genes and their orthologs in closely related species. BMC Bioinformatics.

[24] Hatje K, Keller O, Hammesfahr B, Pillmann H, Waack S, Kollmar M (2011). Cross-species protein sequence and gene structure prediction with fine-tuned Webscipio 2.0 and Scipio. BMC Res. Notes.

[25] Hammesfahr B, Hatje K, Kollmar M, Waack S. Scipio eukaryotic gene identification: Help. 2015. http://www.webscipio.org/help/webscipio#setting.

[26] Pavesi G, Zambelli F, Caggese C, Pesole G (2008). Exalign: a new method for comparative analysis of exon-intron gene structures. Nucleic Acids Res.

[27] Wain HM, Bruford EA, Lovering RC, Lush MJ, Wright MW, Povey S (2002). Guidelines for human gene nomenclature. Genomics.

[28] Vilella AJ, Severin J, Ureta-Vidal A, Heng L, Durbin R, Birney E (2009). EnsemblCompara GeneTrees: Complete, duplication-aware phylogenetic trees in vertebrates. Genome Res.

[29] Altschul SF, Gish W, Miller W, Myers EW, Lipman DJ (1990). Basic local alignment search tool. J Mol Biol.

[30] Eddy SR (2008). A probabilistic model of local sequence alignment that simplifies statistical significance estimation. PLoS Comput Biol.

[31] Eddy SR (2011). Accelerated profile HMM searches. PLoS Comput Biol.

[32] Lovász L, Plummer MD (1986). Matching theory.

[33] Burkard R, Dell’Amico M, Martello S (2012). Assignment problems.

[34] Karp RM, Miller RE, Thatcher JW (1972). Reducibility among combinatorial problems. Complexity of computer computations.

[35] Dalquen DA, Anisimova M, Gonnet GH, Dessimoz C (2012). ALF-a simulation framework for genome evolution. Mol Biol Evol.

[36] Renninger SL, Gesemann MN, Stephan CF (2011). Cone arrestin confers cone vision of high temporal resolution in zebrafish larvae. Eur J Neurosci.

[37] Silva JP, Ushkaryov YA (2010). The latrophilins, “split-personality” receptors. Adv Exp Med Biol.

[38] Harty BL, Krishnan A, Sanchez NE, Schiöth HB, Monk KR (2015). Defining the gene repertoire and spatiotemporal expression profiles of adhesion G protein-coupled receptors in zebrafish. BMC Genomics.

[39] Hatje K, Keller O, Hammesfahr B, Pillmann H, Waack S, Kollmar M (2011). Cross-species protein sequence and gene structure prediction with fine-tuned Webscipio 2.0 and Scipio. BMC Res Notes.

[40] Setta ND, Monteiro-Vitorello CB, Metcalfe CJ, Cruz GMQ, Del Bem LE, Vicentini R, Nogueira FTS, Campos RA, Nunes SL, Turrini PCG, Vieira AP, Ochoa Cruz EA, Corrêa TCS, Hotta CT, de Mello Varani A, Vautrin S, da Trindade AS, de Mendonça Vilela M, Lembke CG, Sato PM, de Andrade RF, Nishiyama MY, Cardoso-Silva CB, Scortecci KC, Garcia AAF, Carneiro MS, Kim C, Paterson AH, Bergès H, D’Hont A, de Souza AP, Souza GM, Vincentz M, Kitajima JP, van Sluys MA (2014). Building the sugarcane genome for biotechnology and identifying evolutionary trends. BMC genomics.

[41] Scherer S (2010). Guide to the human genome.

[42] Shepelev V, Fedorov A (2006). Advances in the exon-intron database (EID). Briefings Bioinf.

[43] Burset M, Seledtsov IA, Solovyev VV (2001). SpliceDB: database of canonical and non-canonical mammalian splice sites. Nucleic Acids Res.

[44] Camacho C, Coulouris G, Avagyan V, Ma N, Papadopoulos J, Bealer K, Madden TL (2009). BLAST+: architecture and applications. BMC Bioinformatics.

[45] Williams G. getorf. MRC Rosalind Franklin Centre for Genomics Research Wellcome Trust Genome Campus. 2002. http://emboss.toulouse.inra.fr/cgi-bin/emboss/help/getorf. Accessed 17 June 2015.

[46] Larkin MA, Blackshields G, Brown NP, Chenna R, McGettigan PA, McWilliam H, Valentin F, Wallace IM, Wilm A, Lopez R, Thompson JD, Gibson TJ, Higgins DG (2007). Clustal W and Clustal X version 2.0.. Bioinformatics.

[47] Sievers F, Wilm A, Dineen D, Gibson TJ, Karplus K, Li W, Lopez R, McWilliam H, Remmert M, Soding J, Thompson JD, Higgins DG (2011). Fast, scalable generation of high-quality protein multiple sequence alignments using Clustal Omega. Mol Syst Biol.

[48] Huson DH, Richter DC, Rausch C, Dezulian T, Franz M, Rupp R (2007). Dendroscope: An interactive viewer for large phylogenetic trees. BMC Bioinformatics.

