# Supplementary material for: The paralog-to-contig assignment problem: high quality gene models from fragmented assemblies
Source: Algorithms Mol Biol. 2016 Feb 24;11:1. doi: 10.1186/s13015-016-0063-y (PMC4765045; doi:10.1186/s13015-016-0063-y)

Additional file 1 — Detailed illustration of the EMS-pipeline

Figure 1 Extended schematic on steps, user-options and input-modes of the EMS-pipeline. The starting points for the three different modes of the EMS-pipeline are illustrated by red, yellow and green dots as are the different input files required for the respective mode. User options are given on the left and right side in yellow. See legend for further explanation. Abbreviations: hMM – hidden Markov Model, TCE - translated coding exon, WGD – whole genome duplication.

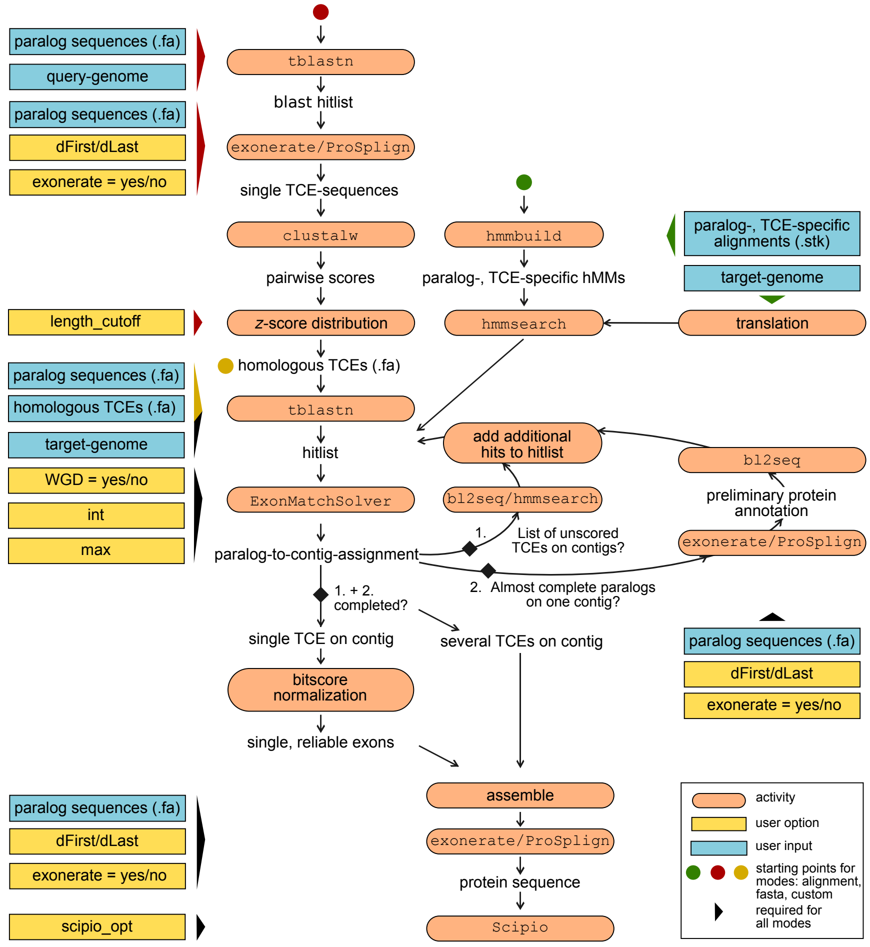

Supplement: Supplementary file 1 — 10.1186/s13015-016-0063-y Detailed illustration of the EMS-pipeline. Extended schematic on steps, user-options and input-modes of the EMS-pipeline. The starting points for the three different modes of the EMS-pipeline are illustrated by red, yellow and green dots as are the different input files required for the respective mode. User options are given on the left and right side in yellow. See legend for further explanation. hMM hidden Markov Model, TCE translated coding exon, WGD whole genome duplication. [file 13015_2016_63_MOESM1_ESM.pdf]
