# Supplementary material for: The paralog-to-contig assignment problem: high quality gene models from fragmented assemblies
Source: Algorithms Mol Biol. 2016 Feb 24;11:1. doi: 10.1186/s13015-016-0063-y (PMC4765045; doi:10.1186/s13015-016-0063-y)
Supplement: Supplementary file 2 — 10.1186/s13015-016-0063-y Phylogenetic tree of arrestin annotations from Scipio. Phylogenetic tree of arrestins as annotated by Scipio in pufferfish together with query orthologs from zebrafish. Scipio was run in sensitive mode (modified options: max_assemble_size = 50000, min_score = 0.1, exhaust_align_size = 50000, region_size = 90000). The alignment and neighbor joining-tree on protein level were built with Clustal Omega 1.2.1 (Sievers et al. 2011 [1]) on all columns of the alignment (A) and on all columns that did not contain any gaps (B). The alternative solutions of Scipio on the same genomic unit may slightly differ in dependence on the query paralog it was retrieved with (indicated by first part of the node label). The zebrafish paralogs are denoted as SAGa,b, ARRB1, ARRB2a,b and ARR3a,b. The solution proposed by the EMS-pipeline is highlighted in green. ARRB1 is situated on scaffold_2476_scaffold_8066. Due to long branch attraction, missing data and sequence divergence, zebrafish and pufferfish orthologs do not always form monophyletic groups. This makes inference of paralog-to-contig assignments based on the phylogenetic tree difficult. The trees were edited and displayed with Dendroscope 2.6.1 (Huson et al. 2007 [2]) [file 13015_2016_63_MOESM2_ESM.pdf]

Additional file 2 — Phylogenetic tree of arrestin annotations from `Scipio`

Figure 1 Phylogenetic tree of arrestins as annotated by `Scipio` in pufferfish together with query orthologs from zebrafish. `Scipio` was run in sensitive mode (modified options: max_assemble_size=50000, min_score=0.1, exhaust_align_size=50000, region_size=90000). The alignment and neighbor joining-tree on protein level were built with `Clustal Omega 1.2.1` [1] on all columns of the alignment (A) and on all columns that did not contain any gaps (B). The alternative solutions of `Scipio` on the same genomic unit may slightly differ in dependence on the query paralog it was retrieved with (indicated by first part of the node label). The zebrafish paralogs are denoted as *SAGa,b*, *ARRB1*, *ARRB2a,b* and *ARR3a,b*. The solution proposed by the EMS-pipeline is highlighted in green. *ARRB1* is situated on `scaffold_2476_scaffold_8066`. Due to long branch attraction, missing data and sequence divergence, zebrafish and pufferfish orthologs do not always form monophyletic groups. This makes inference of paralog-to-contig assignments based on the phylogenetic tree difficult. The trees were edited and displayed with `Dendroscope 2.6.1` [2].

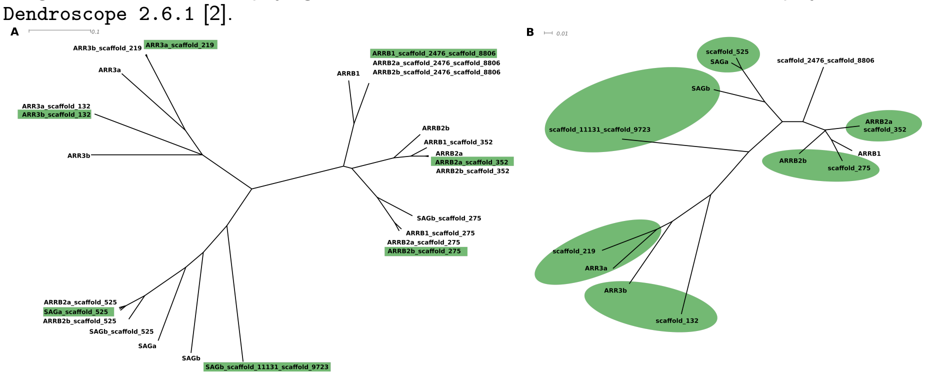

Author details

References

1. Sievers, F., Wilm, A., Dineen, D., Gibson, T.J., Karplus, K., Li, W., Lopez, R., McWilliam, H., Remmert, M., Soding, J., Thompson, J.D., Higgins, D.G.: Fast, scalable generation of high-quality protein multiple sequence alignments using Clustal Omega. Molecular Systems Biology **7**(1) (2011). doi:10.1038/msb.2011.75
2. Huson, D.H., Richter, D.C., Rausch, C., Dezulian, T., Franz, M., Rupp, R.: Dendroscope: An interactive viewer for large phylogenetic trees. BMC Bioinformatics **8**(1), 1–6 (2007). doi:10.1186/1471-2105-8-460
