# Supplementary material for: The paralog-to-contig assignment problem: high quality gene models from fragmented assemblies
Source: Algorithms Mol Biol. 2016 Feb 24;11:1. doi: 10.1186/s13015-016-0063-y (PMC4765045; doi:10.1186/s13015-016-0063-y)
Supplement: Supplementary file 4 — 10.1186/s13015-016-0063-y Table of paralog-to-contig assignments of arrestins in pufferfish. Performance of Scipio and the EMS-pipeline in prediction of arrestin genes in pufferfish. The pufferfish genome FUGU 4.0 (Ensembl) was queried with zebrafish protein sequences (NP_001153294.1, AAH76177.1, AAI52656.1, NP_957418.1 and annotations from Ensembl Zv9). Scipio was run with “cross-species default options” (min_identity = 60, max_move_exon = 6, blat_score = 15, blat_identity = 54, multiple_results, region_size = 10000, exhaust_align_size = 15000, results given in bold) and in a more sensitive mode (modified options: max_assemble_size = 50000, min_score = 0.1, exhaust_align_size = 50000, region_size = 90000). The sensitive Scipio-mode included all hits of the cross-species default options. If scores deviated, these are separated by “/”. As Scipio was run with the multiple_results-option, several hits are occasionally returned; these are indicated by a number in brackets in the paralog-column. The EMS-pipeline was run in “custom-mode” with ProSplign as spliced alignment tool. TCE-numbering refers to the homologous TCE-groups. Hits were considered even if they were partial only. fp false positive, s scaffold. [file 13015_2016_63_MOESM4_ESM.pdf]

Additional file 4 — Table of paralog-to-contig assignments of arrestins in pufferfish

Performance of `Scipio` and the EMS-pipeline in prediction of arrestin genes in pufferfish. The pufferfish genome *FUGU 4.0* (`Ensembl`) was queried with zebrafish protein sequences (NP_001153294.1, AAH76177.1, AAI52656.1, NP_957418.1 and annotations from `Ensembl` *Zv9*). `Scipio` was run with "cross-species default options" (min_identity=60, max_move_exon=6, blat_score=15, blat_identity=54, multiple_results, region_size=10000, exhaust_align_size=15000, results given in bold) and in a more sensitive mode (modified options: max_assemble_size=50000, min_score=0.1, exhaust_align_size=50000, region_size=90000). The sensitive `Scipio`-mode included all hits of the cross-species default options. If scores deviated, these are separated by "/". As `Scipio` was run with the multiple_results-option, several hits are occasionally returned; these are indicated by a number in brackets in the paralog-column. The EMS-pipeline was run in "custom-mode" with `ProSplign` as spliced alignment tool. TCE-numbering refers to the homologous TCE-groups. Hits were considered even if they were partial only. Abbreviations: fp – false positive, s – scaffold.

`Scipio`				EMS-pipeline		
paralog	contig	score	TCEs identified	contig assignment	TCEs included by the `ExonMatchSolver`	TCEs included after post-processing (`ProSplign`)
**SAGa**	s_525	0.426	1-12	s_525	3-12, 14, 16	1-16
**SAGb**	s_525	0.322	5-14, 16	s_11131	6-8, 10-11	6-8, 10, 11
SAGb(1)	s_275	0.151	3-7, 11, 12, 14	s_9723	14, 16	14, 16
SAGb(2)	s_11131	0.127	6-8, 10, 11			
	s_9723	0.052	14, 16			
**ARRB1**	s_352	**0.536**/0.538	3-12, 14-16	s_2476	2-6	1-6
**ARRB1(1)**	s_275	0.44	2-14	s_8806	9-11	9-11
ARRB1(2)	s_2476	0.225	2-6	s_13	8, 14 (fp)	-
	s_8806	0.187	9-11			
**ARRB2a**	s_352	1.000	1-12, 14-16	s_352	2-12, 14-16	1-12, 14-16
**ARRB2a(1)**	s_275	0.527	2-12, 14			
ARRB2a(2)	s_2476	0.167	2-6			
	s_8806	0.150	9-11			
ARRB2a(3)	s_525	0.126	3, 8-12			
**ARRB2b**	s_352	**0.797**/0.819	1-12, 14-16	s_275	3-12, 14, 16	1-12, 14-16
**ARRB2b(1)**	s_275	0.529	2-12, 14			
ARRB2b(2)	s_2476	0.162	2-6			
	s_8806	0.154	9-11			
ARRB2b(3)	s_525	0.152	3, 5-12			
**ARR3a**	s_219	0.457	3-14	s_219	5-12, 14	2-14
ARR3a(1)	s_132	0.367	2-14			
ARR3b	s_132	0.238	2-11	s_132	3-12, 14	2-14
ARR3b(1)	s_219	0.210	5-12			
