# Supplementary material for: The paralog-to-contig assignment problem: high quality gene models from fragmented assemblies
Source: Algorithms Mol Biol. 2016 Feb 24;11:1. doi: 10.1186/s13015-016-0063-y (PMC4765045; doi:10.1186/s13015-016-0063-y)
Supplement: Supplementary file 5 — 10.1186/s13015-016-0063-y Table of paralog-to-contig assignments of latrophilins in cod. Performance of Scipio and the EMS-pipeline in prediction of latrophilin genes in cod. The cod genome gadMor1 (Ensembl) was queried with zebrafish protein sequences (Ensembl GPRCz10). Scipio was run with “cross-species default options” (min_identity = 60, max_move_exon = 6, blat_score = 15, blat_identity = 54, multiple_results, region_size = 10000, exhaust_align_size = 15000). Scipio occasionally returned several hits; these are indicated by a number in brackets in theparalog-column. The EMS-pipeline was run in “custom-mode” with exonerate as spliced alignment tool. The TCE numbering refers to the homologous TCE-groups. Hits were considered even if they were partial only. c contig, fp false positive, GS GeneScaffold, s scaffold. [file 13015_2016_63_MOESM5_ESM.pdf]

Additional file 5 — Table of paralog-to-contig assignments of latrophilins in cod

Performance of `Scipio` and the EMS-pipeline in prediction of latrophilin genes in cod. The cod genome *gadMor1* (`Ensembl`) was queried with zebrafish protein sequences (`Ensembl` *GPRCz10*). `Scipio` was run with "cross-species default options" (min_identity=60, max_move_exon=6, blat_score=15, blat_identity=54, multiple_results, region_size=10000, exhaust_align_size=15000). `Scipio` occasionally returned several hits; these are indicated by a number in brackets in the paralog-column. The EMS-pipeline was run in "custom-mode" with `exonerate` as spliced alignment tool. The TCE-numbering refers to the homologous TCE-groups. Hits were considered even if they were partial only. Abbreviations: c – contig, fp – false positive, GS – GeneScaffold, s – scaffold.

Scipio				EMS-pipeline		
paralog	contig	score	TCEs identified	contig assignment	TCEs included by the ExonMatchSolver	TCEs included after post-processing (exonerate)
ADGRL1a	GS_3732	0.005	1 (fp)	GS_2428	2, 5-7, 9-23, 25	2, 5-9, 11-23, 25
	GS_2428	0.681	2, 5-7, 9, 11-23, 25	c445060	3	3
ADGRL1a(1)	GS_1780	0.354	2-7, 9, 11, 12, 14-20, 22, 23, 25			
ADGRL1b	GS_2428	0.411	2, 5-7, 9-12, 14-18, 20, 22, 23, 25	GS_3850	2, 3, 5-20, 22, 23	2, 3, 5-12, 14-23, 25
ADGRL1b(1)	GS_3850	0.309	2-12, 14, 15, 17, 22			
ADGRL2a	GS_1780	0.643	2-23, 25	GS_1780	2, 3, 5-23, 25	2, 3, 5-23, 25
ADGRL2a(1)	GS_2759	0.425	2-7, 11-17, 19-20, 22, 23, 25			
	GS_1138	0.005	25			
ADGRL2a(2)	GS_871	0.005	1			
	GS_2428	0.316	2, 5-7, 9-12, 14-18, 20-23, 25			
ADGRL2b	GS_1780	0.527	1-23, 25	GS_2759	1 (fp), 2-3, 5-7, 11-17, 19-23, 25	2, 3, 5-7, 11-17, 19-23, 25
ADGRL2b(1)	GS_2759	0.513	2-7, 11-17, 19-23, 25	s10419	18	18
ADGRL2b(2)	GS_3429	0.009	1	c348339	10	10
	GS_2428	0.300	2, 5-7, 9-18, 20, 22, 25			
ADGRL3a	GS_2409	0.007	1 (fp)	GS_4007	2, 3, 5-7, 9-12, 15, 16, 19, 21-23, 25	1-3, 5-7, 9-12, 15, 16, 19, 21-23, 25
	GS_3151	0.463	2, 3, 5-14, 16, 17, 19, 23-25	GS_4123	8 (fp), 17, 18 (fp)	17 (fp, belongs to CELSR1b)
ADGRL3a(1)	GS_4007	0.453	1-7, 9-12, 15, 16, 19, 21-25	c408339	13	13
				c136327	14	14
ADGRL3b	GS_3151	0.533	2, 3, 5-7, 9, 10, 13, 14, 16, 17, 19, 23-25	GS_3151	2, 3, 5-12, 15-17, 19, 23, 25	2, 3, 5-10, 13-17, 19, 23-25
ADGRL3b(1)	GS_4007	0.402	2-7, 9, 10, 15, 16, 19, 21-23, 25	c12548	21, 22	21, 22
				c493181	18	18
